# Clinical analysis of the serum muscle enzyme spectrum of patients with newly diagnosed Sheehan’s syndrome

**DOI:** 10.1097/MD.0000000000030834

**Published:** 2022-09-30

**Authors:** Hongjiao Gao, Qiao Xiang, Jindie Li, Meng Yu, Yalin Lan, Junqiang Ba, Yan Liu, Haoming Tian

**Affiliations:** a Department of Endocrinology and Metabolism, West China Hospital of Sichuan University, Chengdu, China; b Department of Endocrinology, the Third Affiliated Hospital of Zunyi Medical University (The First People’s Hospital of Zunyi), Zunyi, China; c Department of Laboratory Medicine, the Third Affiliated Hospital of Zunyi Medical University, Zunyi, China.

**Keywords:** creatine kinase, hyponatremia, muscle enzyme, rhabdomyolysis, Sheehan’s syndrome

## Abstract

We investigated the factors associated with serum muscle enzyme elevation in patients with Sheehan’s syndrome. A total of 48 patients who were newly diagnosed with Sheehan’s syndrome were included and divided into 3 groups: Group 1, creatine kinase (CK) ≥ 1000 U/L; Group 2, 140 < CK < 1000 U/L; and Group 3, CK ≤ 140 U/L. Differences in serum muscle enzymes, serum electrolytes, blood glucose and hormones were compared among the 3 groups. A Spearman correlation analysis and multiple linear regression analysis were performed on serum muscle enzymes and the other variables. Four patients in Group 1 underwent electromyography. Fourteen, 26 and 8 patients were divided into Group 1, Group 2, and Group 3, respectively. The levels of plasma osmolality, serum sodium, free triiodothyronine (FT3) and free thyroxine (FT4) in Group 1 were lower than those in Group 3 at admission (*P* < .05). There were significant differences in CK, CK-MB, aspartate aminotransferase, lactate dehydrogenase, and alpha-hydroxybutyrate dehydrogenase among the three groups (*P* < .05). CK was correlated with serum sodium (*r* = −0.642, *P* < .001), serum potassium (*r* = −0.29, *P* = .046), plasma osmolality (*r* = −0.65, *P* < .001), FT3 (*r* = −0.363, *P* = .012), and FT4 (*r* = −0.450, *P* = .002). Moreover, creatine kinase isoenzyme-MB (CK-MB) was correlated with serum sodium (*r* = −0.464, *P* = .001) and plasma osmolality (*r* = −0.483, *P* < .001). The multiple linear regression showed that serum sodium was independently and negatively correlated with CK (*r* = −0.352, *P* = .021). The electromyogram results supported the existence of myogenic injury. Sheehan’s syndrome is prone to be complicated by nontraumatic rhabdomyolysis, with both a chronic course and acute exacerbation. Serum muscle enzymes should be routinely measured. For patients with CK levels > 1000 U/L, a CK-MB/CK ratio < 6% can be a simple indicator to differentiate rhabdomyolysis from acute myocardial infarction. Abnormal serum muscle enzymes observed in Sheehan’s syndrome may be associated with hypothyroidism and with hyponatremia in particular.

## 1. Introduction

Sheehan’s syndrome is an important cause of anterior pituitary hypofunction in developing countries^[1,2]^ and is caused by ischemic necrosis of the pituitary gland after severe postpartum hemorrhage.^[2–5]^ It can manifest as hypofunction of target glands, including the thyroid gland, adrenal glands and gonad, and it is often accompanied by various symptoms and a delayed diagnosis.^[3]^ This disorder is not rare in areas with underdeveloped economies and transportation, which may greatly affect the physical and mental health of patients.

The muscle enzyme spectrum is composed of aspartate amino-transferase (AST), lactate dehydrogenase (LDH), α-hydroxybutytate (α-HBDH), creatine kinase (CK) and its isoenzyme CK-MB.^[6]^ CK-MB mainly exists in the myocardium, and it comprises approximately 14% of the total CK in the myocardium. CK-MB content is low in tissues other than skeletal muscle.^[7,8]^ Muscle enzymes exhibit a certain value in the diagnosis of acute myocardial infarction (AMI) and for the assessment of the onset time, involved area, reperfusion and reinfarction of the tissue; however, they can also be increased when the skeletal muscle is injured. To avoid false-positive CK-MB elevation originating from skeletal muscle, the CK-MB/CK ratio was introduced to improve the specificity of CK-MB elevation for AMI.^[9]^

Patients with Sheehan’s syndrome usually have an abnormal spectrum of serum muscle enzymes,^[10]^ which can also be observed in various diseases, including AMI,^[11]^ metabolic or inflammatory myopathy,^[12,13]^ and rhabdomyolysis (RM),^[14]^ among other disorders. For example, as a member of the serum muscle enzyme group, CK is often markedly elevated in many endocrine disorders,^[15,16]^ which should be treated with caution. Furthermore, clinicians in primary care may misdiagnose Sheehan’s syndrome as AMI due to an insufficient recognition of the disorder; conversely, severely abnormal muscle enzymes may also be associated with acute kidney injury.^[17]^

RM is a pathological syndrome caused by skeletal muscle (striated muscle) cell injury that affects the integrity of cell membranes and causes the release of toxic components within the cells into the blood circulation.^[18]^ Previous case reports have suggested that RM should be considered when Sheehan’s syndrome is accompanied by a significant increase in serum muscle enzymes^[10,19,20]^; however, there were no electromyographic results.

In this study, we found that 40 out of the 48 patients with newly diagnosed Sheehan’s syndrome had an abnormal spectrum of serum muscle enzymes. Among those patients, 14 patients showed CK levels over 1000 U/L, and 4 patients in Group 1 underwent electromyogram. In addition, their serum muscle enzyme spectrum was dynamically observed after admission. Our study could be conducive to research on the etiology, pathogenesis, progression and prognosis of muscle enzyme elevation in those patients, and it could be of clinical significance for reducing misdiagnosis, reducing unnecessary examinations and improving prognosis.

## 2. Methods

### 2.1. Patients

A total of 70 patients with Sheehan syndrome were admitted to the Endocrinology Department of the Third Affiliated Hospital of Zunyi Medical University from December 2009 to August 2020, and among them, 48 were newly diagnosed and had never taken thyroxine or glucocorticoid replacement therapy. All of the included cases met the following classic diagnostic criteria for Sheehan’s syndrome^[5]^: the patient had a clear history of postpartum hemorrhage and severe hypotension or shock requiring blood transfusions or rehydration; the patient developed agalactia after delivery and was unable to resume normal menstruation; the patient developed different degrees of anterior pituitary hypofunction; and the patient’s computer tomography or magnetic resonance imaging results showed partial or complete empty sella. The exclusion criteria were as follows: the patient suffered from AMI, myocarditis or severe liver, kidney or brain diseases; the patient had a recent history of muscle injury; the patient suffered from myositis, progressive muscular dystrophy, myotonic myopathy, mitochondrial myopathy, alcoholism or neurological diseases; and the patient had recently taken medication that had a clear effect on CK levels, such as lipid-lowering medications including statins and fibrates, analgesics, sedatives and macrolides, among other medications. CK ≥ 1000 U was considered the diagnostic threshold for RM,^[21]^ and 140 U/L was the upper limit of normal for the measured CK provided by our laboratory, and we grouped them accordingly. The patients who were finally included in the study were divided into 3 groups (according to the CK levels measured at admission): Group 1, CK ≥ 1000 U/L; Group 2, 140 < CK < 1000 U/L; and Group 3, normal CK levels (≤140 U/L).

### 2.2. Variables

The data of 1 patient who was hospitalized in December 2009 were retrospectively retrieved. For the remaining 47 patients who were hospitalized after 2011, their venous blood samples were immediately collected after admission to determine levels of the muscle enzyme spectrum (including CK, CK-MB, AST, LDH, and HBDH), serum electrolytes (including potassium, sodium, chloride, calcium, phosphorus, and magnesium), blood glucose, renal function indicators (including urea nitrogen and creatinine), free triiodothyronine (FT3), free thyroxine (FT4), thyroid stimulating hormone, and cortisol. The effective plasma osmolality was calculated according to the following formula: effective plasma osmolality = 2 × (Na^+^+K^+^)+blood glucose (mmol/L).^[22]^ In Group 1, serum myoglobin was measured in six patients and four patients underwent electromyography. For patients who possessed CK-MB levels that were more than twice the upper limit of normal, their serum cardiac troponin T levels were measured because the diagnostic value of troponin as a marker of myocardial necrosis is higher than that of CK-MB or myoglobin^[23]^; additionally, their conventional 12-lead surface electrocardiogram values were measured via a bedside electrocardiogram recorder. If the abovementioned indicators were abnormal, observations of changes in the serum muscle enzyme spectrum were initiated from the second day of admission until they returned to normal. Informed consent was obtained from all of the subjects, and this study was approved by the Ethics Committee of the First People’s Hospital of Zunyi (no. 2012-1-091).

### 2.3. Statistical analysis

Continuous variables are presented as the mean and standard deviation if they were normally distributed; otherwise, variables are presented as the median and interquartile range, while categorical variables are presented as frequencies and percentages. The one-way analysis of variance test or Kruskal–Wallis *H* test was used to compare differences in the continuous variables between groups (according to the data distribution). A Spearman rank correlation analysis and multiple linear regression analysis were performed on serum muscle enzymes and other variables in patients with Sheehan’s syndrome. A *P* value < .05 was considered to be statistically significant. All of the statistical analyses were performed using SPSS version 20.0 (IBM Corp, Armonk, NY).

## 3. Results

As described above, a total of 48 patients with Sheehan’s syndrome were included in this study. The age of onset ranged from 17 to 45 years, with an average age of 30.44 ± 6.64-years-old. The age at diagnosis ranged from 28 to 78 years, with an average age of 49.21 ± 10.34-years-old. The average time from the onset to diagnosis was 18 years, ranging from 1 to 10 years for 10 (20.83%) patients, from 21 to 30 years for 16 (33.3%) patients and over 30 years for 5 (10.42%) patients.

Fourteen, 26 and 8 patients were divided into Group 1, Group 2, and Group 3, respectively.

### 3.1. Comparison of baseline characteristics at admission between groups

The levels of plasma osmolality, serum sodium, FT3 and FT4 in Group 1 were significantly lower than those in Group 3 (*P* < .05), whereas the age at diagnosis, time from onset to diagnosis and levels of serum potassium, serum calcium, blood glucose and thyroid stimulating hormone were not significantly different between Group 1 and Group 3 (*P* > .05). The FT4 levels of Group 2 were significantly lower than those of Group 3, and the serum sodium levels of Group 1 were significantly lower than those of Group 2 (*P* < .05). Cortisol levels of Group 1 were significantly lower than those of Group 2 or Group 3 (*P* < .05) (Table 1).

**Table 1:** Comparison of baseline characteristics at admission between groups.

	Group 1	Group 2	Group 3	*P* value	*P* (1vs2)	*P* (1vs3)	*P* (2vs3)
Age at diagnosis (years old)	49.57 ± 10.30	49.38 ± 10.40	48.00 ± 11.50	.938	/	/	/
Time from onset to diagnosis (yr)	18.50 ± 10.03	18.46 ± 10.06	17.88 ± 12.41	.989	/	/	/
Serum sodium (mmol/L)	117.99 ± 10.97	127.85 ± 10.83	139.95 (5.32)	.001	.036	.001	.121
Serum potassium (mmol/L)	3.41 ± 0.43	3.68 ± 0.58	3.84 ± 0.52	.149	/	/	/
Serum calcium (mmol/L)	2.00 ± 0.24	2.14 (0.22)	2.04 ± 0.29	.136	/	/	/
Effective plasma osmolality (mmol/L)	246.62 ± 21.32	267.20 ± 22.68	293.45 (6.31)	<.001	.063	<.001	.057
Blood glucose (mmol/L)	3.82 ± 1.58	4.20 (2.25)	4.50 (3.98)	.450	/	/	/
FT3 (pmol/L)	2.24 (0.95)	2.48 ± 0.90	3.22 ± 1.03	.027	.573	.022	.189
FT4 (pmol/L)	2.86 ± 2.52	2.88 ± 1.80	5.90 ± 1.90	.002	.981	.002	.001
TSH (μIU/mL)	1.85 (2.05)	1.79 (1.97)	3.60 ± 3.24	.406	/	/	/
Cortisol (nmol/L)	29.73 (23.81)	60.92 (58.92)	103.31 (125.12)	.003	.045	.001	.031

Group 1, CK ≥ 1000 U/L; Group 2, 140 < CK < 1000 U/L; Group 3, normal CK levels (≤140 U/L). Reference range: FT3, 3.8–6 pmol/L; FT4, 7.86–14.4 pmol/L; TSH, 0.34–5.6 µIU/mL; cortisol, 185–624 nmol/L.

CK = creatine kinase, FT3 = free triiodothyronine, FT4 = free thyroxine, TSH = thyroid stimulating hormone.

### 3.2. Comparison of serum muscle enzymes between groups

In addition to CK, there were also significant differences in CK-MB, AST, LDH, and HBDH among the 3 groups. In particular, the pairwise comparison indicated that the CK-MB/CK ratio was significantly different between every 2 groups (*P* < .01), which decreased with increasing CK (Table 2).

**Table 2:** Comparison of serum muscle enzymes between groups.

	Group 1	Group 2	Group 3	*P* value	*P* (1vs2)	*P* (1vs3)	*P* (2vs3)
CK (U/L)	2270.86 (2202.40)	294.55 (470.33)	68.74 ± 30.29	<.001	.008	<.001	<.001
CK-MB (U/L)	60.95 (83.18)	13.30 (17.05)	12.07 ± 3.46	<.001	1.000	<.001	<.001
AST (U/L)	94.50 (65.79)	53.50 (35.35)	29.00 (29.23)	<.001	.006	<.001	<.001
LDH (U/L)	439.00 (286.05)	247.58 ± 71.12	193.99 ± 59.76	<.001	.001	<.001	<.001
HBDH (U/L)	285.05 (245.13)	185.78 ± 60.92	147.94 ± 36.13	<.001	.001	<.001	<.001
CK-MB/CK (%)	2.59 (1.28)	4.61 (3.53)	19.67 ± 7.57	<.001	.008	<.001	<.001

Group 1, CK ≥ 1000 U/L; Group 2, 140 < CK < 1000 μ/L; Group 3, normal CK levels (≤140 U/L). Reference range: CK, 26–140 U/L; CK-MB, 0–25 U/L; AST, 10–40 U/L; LDH, 104–245 U/L; HBDH, 90–180 U/L.

AST = aspartate amino-transferase, CK = creatine kinase, CK-MB = creatine kinase isoenzyme-MB, HBDH = α-hydroxybutytate, LDH = lactate dehydrogenase.

Myoglobin was measured in 6 patients with increased CK, with a median level of 267.7 ng/mL (interquartile range: 463.2 ng/mL, reference range: 28–72 ng/mL).

### 3.3. Spearman rank correlation analysis and multiple linear regression analysis

As indicated by the Spearman correlation analysis, CK was negatively correlated with serum sodium (*r* = −0.642, *P* < .001), serum potassium (*r* = −0.29, *P* = .046), plasma osmolality (*r* = −0.65, *P* < .001), FT3 (*r* = −0.363, *P* = .012), and FT4 (*r* = −0.450, *P* = .002). Additionally, CK-MB levels were negatively correlated with serum sodium (*r* = −0.464, *P* = .001) and plasma osmolality (*r* = −0.483, *P* < .001) (Table 3).

**Table 3:** Spearman correlation analysis.

	CK (U/L)		CK-MB (U/L)	
	*r*	*P*	*r*	*P*
Age at diagnosis (years old)	−0.050	.736	−0.097	.513
Time from onset to diagnosis (yr)	0.032	.831	0.105	.479
Serum sodium (mmol/L)	−0.642	<.001	−0.483	<.001
Serum potassium (mmol/L)	−0.290	.046	−0.087	.559
Serum calcium (mmol/L)	−0.200	.184	−0.286	.054
Effective plasma osmolality (mmol/L)	−0.650	<.001	−0.464	.001
Blood glucose (mmol/L)	−0.282	.054	−0.231	.118
FT3 (pmol/L)	−0.363	.012	−0.213	.150
FT4 (pmol/L)	−0.0450	.002	−0.162	.277
TSH (μIU/mL)	−0.132	.376	−0.007	.965
Cortisol (nmol/L)	−0.554	<.001	−0.484	.001

AST = aspartate amino-transferase, CK = creatine kinase, CK-MB = creatine kinase isoenzyme-MB, FT3 = free triiodothyronine, FT4 = free thyroxine, HBDH = α-hydroxybutytate, LDH = lactate dehydrogenase, *r* = Spearman’s correlation coefficient, TSH = thyroid stimulating hormone.

When considering CK as the dependent variable, we incorporated 7 independent variables (including time from onset to diagnosis, serum sodium, serum potassium, serum calcium, blood glucose, FT4, and cortisol) into the multiple linear regression model. The obtained regression equation proved to be statistically significant (*P* = .001). The variance inflation factor for all of the included independent variables was less than 5, thus indicating that there was no multicollinearity among the independent variables. Only serum sodium was found to be independently and negatively correlated with CK (*r* = −0.352, *P* = .021) (Table 4).

**Table 4:** Multiple linear regression analysis.

	Standardized coefficient	*P* value	VIF
Time from onset to diagnosis	0.095	.505	1.399
Serum sodium	−0.352	.021	1.473
Serum potassium	−0.094	.472	1.168
Serum calcium	0.077	.580	1.336
Blood glucose	−0.147	.271	1.200
FT3	−0.217	.177	1.721
FT4	−0.172	.302	1.873
Cortisol	−0.249	.100	1.510
Constant	/	.000	/

Creatine kinase was considered as the dependent variable, and time from onset to diagnosis, serum sodium, serum potassium, serum calcium, blood glucose, FT4, and cortisol were considered as the independent variables.

FT4 = free thyroxine, VIF = variance inflation factor.

### 3.4. Electromyogram

**Figure 1.**
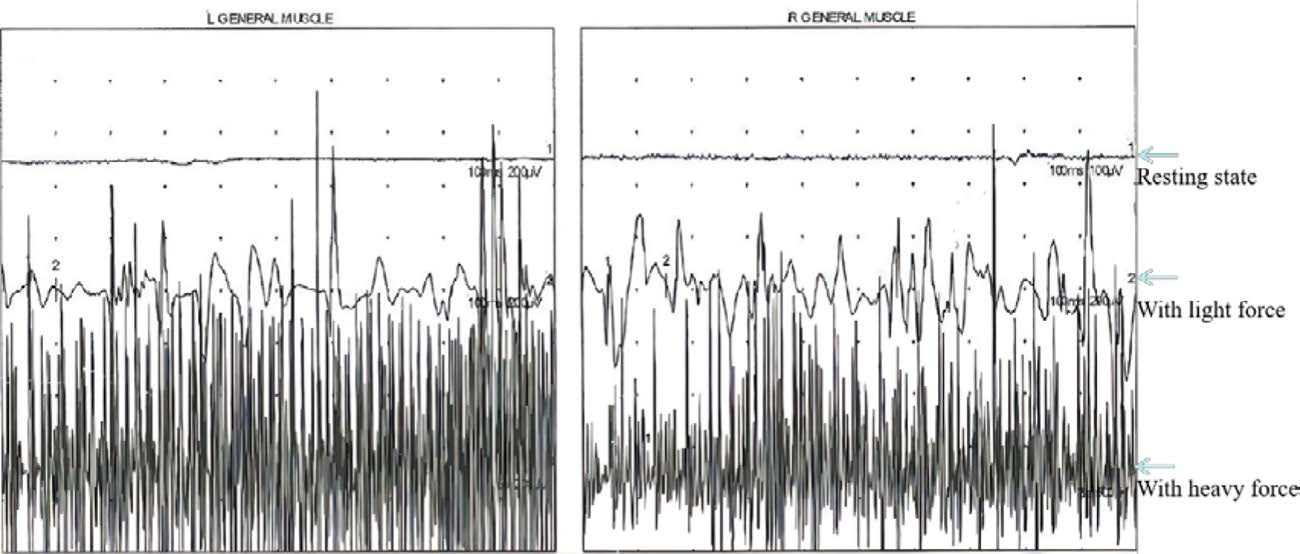
Results of the electromyogram. Concentric needle electromyography of the quadriceps femoris, tibialis anterior muscle and gastrocnemius muscle of both lower limbs was performed. The following features could be seen: the insertion potential was normal, with no denervation potential; there was an increase in multiphase potential, with a reduced average time course and decreased average amplitude; and an interference phase was shown with heavy force. The electromyogram of the examined muscles on both lower limbs showed abnormalities related to myogenic injury.

Four patients underwent electromyography, all of whom showed features consistent with myogenic injury. Figure 1 shows the results of one patient who underwent a concentric needle electromyography of the quadriceps femoris, tibialis anterior muscle and gastrocnemius muscle of both lower limbs.

## 4. Discussion

Sheehan’s syndrome is currently rare in developed countries and in developing countries that possess convenient transportation methods. However, Guizhou Province in China is a mountainous area, and the incidence of Sheehan’s syndrome in this region was relatively high, due to inconvenient transportation and relatively poor medical conditions 20 years ago. In our study, 35 (73%) of the 48 newly diagnosed patients had their onset of this disorder before 2000, whereas only 3 (6%) patients had their onset after 2010, which suggests that with economic development and improvement in medical and health conditions in China, the incidence of the syndrome has been declining. Previous reports on Sheehan’s syndrome accompanied by an abnormal muscle enzyme spectrum have been mostly focused on individual cases, and a comprehensive analysis is indeed clinically important to help determine the influential factors of abnormal myocardial enzymes, which may remind clinicians that they need to differentiate it from AMI, which can help in guiding clinical treatment.

For patients with Sheehan’s syndrome accompanied by abnormal myocardial enzymes, AMI should be considered if two of the following three criteria are met: the patient has a clinical history of ischemic chest pain; the patient’s electrocardiogram shows dynamic changes; and serum markers of myocardial necrosis show dynamic changes in concentration. None of the included patients met the above criteria. Although CK-MB levels can be increased in both AMI and skeletal muscle-related diseases, the CK-MB/CK ratio is usually less than 6% with skeletal muscle damage or noncardiac causes but more than 6% with myocardial injury, which can be used for differential diagnosis.^[9,24]^ Consistently, in Group 1 of our patients (CK ≥ 1000 U/L), the CK-MB/CK ratio ranged from 1.74% to 5.82%, thus suggesting that the lesion was associated with skeletal muscle and that RM should be considered.

The main reasons for RM include trauma, ischemia, infection, drugs, toxins and metabolic disorders, among other disorders. The clinical manifestations of RM can range from asymptomatic increases in creatine phosphokinase to severe acute renal failure and hypovolemic shock.^[25]^ Chemical causes account for the majority of RM, but the characteristics of RM can be different.^[26]^ CK has a long half-life period, and its elevation is an important marker of muscle cell membrane destruction, which can correctly reflect muscle injury with high sensitivity and serve as the most specific indicator for diagnosing RM.^[27]^ RM syndrome should be considered when CK levels exceed 1000 U/L or are more than 5 times the upper limit of normal if AMI, shock, end-stage renal disease or other myogenic injury has been excluded.^[28–30]^ Although an increase in serum myoglobin concentration or myoglobinuria are also signs of skeletal muscle or myocardial injury, the sensitivity is not high due to the short half-life period of myoglobin (only 2–3 hours), and a negative result cannot yet completely rule out RM.^[31–34]^ The electromyogram is also important for assessing whether the injury is a myogenic injury, and the electromyogram results of patients that were observed in our study conformed to the changes in RM.^[35]^

It has been reported that the occurrence of RM in patients with Sheehan’s syndrome was associated with severe hyponatremia,^[10,19]^ which is also supported by our regression analysis results. Hyponatremia is the most common form of electrolyte disorder in Sheehan’s syndrome, in which the pathogenesis is mainly caused by severe hypothyroidism and glucocorticoid deficiency and involves improper secretion of antidiuretic hormone and hypovolemia. Patients with CK levels exceeding 1000 U/L can have varying degrees of anorexia, nausea and even vomiting, which further aggravates hyponatremia. In patients with Sheehan’s syndrome, the secretion of both cortisol and aldosterone can be reduced due to insufficient ACTH secretion, thus weakening the sodium-retaining and potassium-excreting effects; thus, hypokalemia does not easily occur, whereas severe patients may still develop hypokalemia due to anorexia and vomiting. If there is severe hypokalemia, muscles cannot release enough intracellular potassium to dilate blood vessels when they contract, thus resulting in deficient blood supply to skeletal muscles, adenosine triphosphate depletion in muscle cells and the overload of intracellular calcium, which can cause muscle spasms, ischemic necrosis and RM.^[36]^

CK elevation is associated with thyroid function. When thyroid hormone levels are low, glycolysis and oxidative phosphorylation can be downregulated, followed by a decrease in adenosine triphosphate, as well as CK accumulation and leakage from cells; moreover, a decrease in CK clearance is also an important reason for CK elevation.^[15]^ In our study, CK was negatively correlated with FT3 and FT4, which supports the view that abnormal muscle enzymes in patients with Sheehan’s syndrome are associated with hypothyroidism.

We also performed an analysis on patients with primary hypothyroidism who were hospitalized during the same time period, wherein the CK and CK-MB levels of these patients were revealed to be lower than those of patients with Sheehan’s syndrome (data not shown). This also suggests that there are other factors accounting for RM occurrence in patients with Sheehan’s syndrome in addition to cell-damaging myxedema. In view of the fact that CK was more highly correlated with serum sodium in our study, we assume that severe hyponatremia may contribute to the intracellular edema of skeletal muscles with preexisting lesions, which can cause swelling and rupture of muscle cells, thus leading to the massive release of muscle enzymes.

Although hypoglycemia can also lead to muscle enzyme abnormalities in clinical settings,^[37]^ we observed no significant differences in blood glucose among the 3 groups. However, we still noticed that blood glucose levels in Group 1 (CK ≥ 1000 U/L) were lower than those in Group 2 (140 < CK < 1000 U/L), and the latter values were lower than blood glucose levels in Group 3 (CK ≤ 140 U/L), which may be explained by the relatively small sample size.

Acute kidney injury is observed in 10% to 33% of patients with RM. The lowest abnormal level of CK associated with acute kidney injury is 5000 U/L.^[38,39]^ Acute kidney injury caused by RM is associated with myoglobin. When rhabdomyocytes are injured, myoglobin is released into the blood, which can promote an increase in nitric oxide, endothelin, tumor necrosis factor, adenosine, platelet activating factor and complement, among other factors. These substances can stimulate renal vasoconstriction, are deposited and block the distal renal tubules and directly exert cytotoxicity via endocytosis in the proximal renal tubules.^[40]^ Acute kidney injury in Sheehan’s syndrome is not very common, with few cases having been reported in the literature, most of which were caused by RM.^[20,41–43]^ In our study, 4 (29%) out of the 14 patients in Group 1 (CK ≥ 1000 U/L) showed only mild renal dysfunction with increased myoglobin. When regarding the reason why increased CK in Sheehan’s syndrome is not accompanied by a high incidence of severe kidney damage, we assume that it is related to the chronic course of Sheehan’s syndrome, and myoglobin may have been gradually metabolized before producing a relatively high blood concentration.

The muscle enzymes of all of the included patients gradually returned to normal after the patients were given glucocorticoid or thyroid hormone replacement therapies, and fluid and electrolyte disorders were corrected. Even for one patient with a CK level at 54,144 U/L and a CK-MB level at 1111 U/L, her CK and CK-MB levels decreased to 359.5 and 14.3 UL, respectively, on the 10th day after admission. During the course of treatment, the CK levels of some patients increased again, which was considered to be related to hypokalemia^[44]^ but may also be explained by the occurrence of reperfusion injury. Patients with Sheehan’s syndrome who have been in states of low blood pressure, low perfusion for long time periods and a large amount of fluid infusion after admission may suffer from reperfusion damage to the striated muscle, which requires further basic research studies to confirm.

Limitations in our study should be noted. For example, the retrospective nature of our study with a long time period of enrollment may have introduced a potential selection bias. Additionally, some unknown variables that may be potentially important influential factors of serum muscle enzymes were not included in our analysis due to data limitations. Moreover, our study was based on data from a single center with a relatively small sample size. However, when considering that Sheehan’s syndrome is often undiagnosed or misdiagnosed in clinical settings due to a lack of recognition or difficulty in confirming the diagnosis (the average time from onset to diagnosis was 18 years in our study), the current sample size was acceptable, and more evidence from prospective clinical studies with larger sample sizes from other institutions or populations and good designs is expected to verify our findings.

In summary, Sheehan’s syndrome is often accompanied by increased CK levels due to the chronic injury of striated muscles and is mainly associated with hypothyroidism and glucocorticoid deficiency. Severe hyponatremia can contribute to RM. For patients with CK levels exceeding 1000 U/L, RM should be considered, and a CK-MB/CK ratio of less than 6% can be used to differentiate RM from AMI. Glucocorticoid and thyroid hormone replacement therapies should be started as soon as possible for treating Sheehan’s syndrome. In addition, muscle enzymes of some patients can initially increase but then gradually decrease during treatment, and dynamic monitoring is required under this situation.

## Acknowledgment

The statistical analysis of this paper was conducted under the guidance of Litao Huang (master), Program Design and Statistics Office, Department of Clinical Research Management, West China Hospital, Sichuan University.

## Author contributions

**Conceptualization:** Yan Liu, Haoming Tian.

**Data curation:** Qiao Xiang, Jindie Li.

**Formal analysis:** Qiao Xiang.

**Methodology:** Meng Yu.

**Project administration:** Junqiang Ba.

**Software:** Qiao Xiang.

**Supervision:** Haoming Tian.

**Writing – original draft:** Hongjiao Gao.

**Writing – review & editing:** Hongjiao Gao.
